# Functional performance of a bi-layered chitosan-nano-hydroxyapatite osteochondral scaffold: a pre-clinical *in vitro* tribological study

**DOI:** 10.1098/rsos.230431

**Published:** 2024-01-10

**Authors:** Raelene M. Cowie, Laura Macri-Pellizzeri, Jane McLaren, William J. Sanderson, Reda M. Felfel, Colin A. Scotchford, Brigitte E. Scammell, David M. Grant, Virginie Sottile, Louise M. Jennings

**Affiliations:** ^1^ Institute of Medical and Biological Engineering, University of Leeds, Leeds, UK; ^2^ Academic Unit Translational Medical Sciences, School of Medicine, University of Nottingham, Nottingham, UK; ^3^ Academic Unit Injury, Recovery and Inflammation Sciences (IRIS), School of Medicine, University of Nottingham, Nottingham, UK; ^4^ Advanced Materials Research Group, Faculty of Engineering, University of Nottingham, Nottingham, UK; ^5^ Department of Mechanical and Aerospace Engineering, Faculty of Engineering, University of Strathclyde, Glasgow, UK; ^6^ Department of Molecular Medicine, University of Pavia, Pavia, Italy; ^7^ Physics Department, Faculty of Science, Mansoura University, Mansoura, Egypt

**Keywords:** chitosan, tribology, osteochondral graft, natural knee joint, joint simulation

## Abstract

Osteochondral grafts are used for repair of focal osteochondral lesions. Autologous grafts are the gold standard treatment; however, limited graft availability and donor site morbidity restrict use. Therefore, there is a clinical need for different graft sources/materials which replicate natural cartilage function. Chitosan has been proposed for this application. The aim of this study was to assess the biomechanics and biotribology of a bioresorbable chitosan/chitosan-nano-hydroxyapatite osteochondral construct (OCC), implanted in an *in vitro* porcine knee experimental simulation model. The OCC implanted in different surgical positions (flush, proud and inverted) was compared to predicate grafts in current clinical use and a positive control consisting of a stainless steel graft implanted proud of the cartilage surface. After 3 h (10 800 cycles) wear simulation under a walking gait, subsidence occurred in all OCC samples irrespective of surgical positioning, but with no apparent loss of material and low meniscus wear. Half the predicate grafts exhibited delamination and scratching of the cartilage surfaces. No graft subsidence occurred in the positive controls but wear and deformation of the meniscus were apparent. Implanting a new chitosan-based OCC either optimally (flush), inverted or proud of the cartilage surface resulted in minimal wear, damage and deformation of the meniscus.

## 1. Introduction

With an increase in the prevalence of focal chondral or osteochondral lesions in the knee, particularly in younger patients who may not be suitable candidates for knee replacement, there is a clinical need for alternative treatments [1,2]. Osteochondral grafts are one possible option for management of focal osteochondral defects/lesions. Osteochondral grafts derived from a variety of sources have been used in cartilage regeneration with differing clinical outcomes. Autologous tissue has been shown to give good results but there is a limit to the number of grafts which can be harvested, issues with subsequent donor site morbidity, and difficulty matching the geometry of graft and host tissue. Allogeneic tissue can overcome some of these issues but there still remain concerns associated with tissue availability and the possible transmission of pathogens [1,3]. This has led to the development of natural and synthetic polymers for use as osteochondral grafts, a number of which are in current clinical use [4]. Natural polymers may include gelatin, collagen, alginate, chitosan, hyaluronic acid, glycosaminoglycan, starch or bacterial polymers; synthetic materials including polylactic acid, polycaprolactone, polyethyleneglycol and polyglycolic acid have also been used in cartilage regeneration [3–6]. Grafts may be designed to degrade over time as tissue remodels; however, the degradation rate must be tuned and degradation products should be non-toxic [7]. The readily available supply of polymer scaffolds and the potential to tune their structure and composition make them desirable for use in osteochondral repair; however, to function effectively, they should replicate the tribological and mechanical characteristics of cartilage. Healthy articular cartilage provides support for joint contact forces and a bearing surface with low friction and wear. These important functional characteristics rely on its multiphasic nature, which predominantly consists of a solid phase (porous elastic solid matrix) and fluid phase (interstitial fluid) [8,9]. It is well recognized that cartilage exhibits a range of complex characteristics and mechanical behaviours, and the challenge is to create an osteochondral intervention that replicates these while providing the often overlooked tribological properties of low wear and friction that are the primary function of cartilage [10].

Investigation of osteochondral grafts prior to their use in the clinic is primarily carried out through uniaxial biomechanical studies, toxicity tests and biocompatibility studies undertaken either *in vitro* or in small animal models such as rodents or rabbits [11]. In addition, *in vivo* large animal studies may be conducted; however, their high cost and complex logistics represent a limiting factor for many pre-clinical investigations. Functional simulation to replicate the loading and motion of the grafts *in situ* carried out in a carefully controlled laboratory environment has been used in a limited number of studies to investigate tribological and biomechanical function of osteochondral grafts. These techniques have only recently been developed and not yet been widely adopted but have the potential to reduce the number of *in vivo* animal models needed [12–14]. In this study, a porcine knee model was used consistent with previous functional simulation studies of osteochondral allografts [13,14]. In choosing the tissue source, the joint size, cartilage and trabecular bone thickness were considered and porcine tissue most closely matched the human knee [15]. While it is recognized that human tissue may better replicate the host environment and the loading and motion the grafts will be subjected to *in vivo*, tissue from healthy animals that is readily available, of consistent quality and easy to source may better represent knees with a defined focal lesion in which an early intervention may be considered.

This study investigated a new chitosan-based osteochondral construct (OCC) showing biomimetic features and resistance to delamination [16,17]. Chitosan is a polysaccharide abundantly available in the shells of arthropods and cell walls of fungi which is extracted by deacetylation of natural chitin [18]. Chitosan gels have been shown to have low cytotoxicity [19]. When used as an osteochondral graft, its ability to support the attachment and proliferation of mesenchymal stem cells, resorbable nature and the similarity between the structure of chitosan and that of glycosaminoglycans, a key component of the cartilage matrix, make chitosan scaffolds desirable for use in cartilage repair [17,18,20]. Investigations into the mechanical properties of chitosan scaffolds have shown full recovery to their initial geometry following compression, the potential for high porosity and a tuneable pore size. However, under uniaxial compression, even with the addition of nano-hydroxyapatite, a compressive strength less than 1 MPa, several orders of magnitude less than that of cancellous bone, has been measured [16,17]. The tribology of chitosan coatings grafted to both polymer and metal surfaces has been studied. Coating a substrate with chitosan or chitosan brushes has been shown to improve wettability and decrease the coefficient of friction against polyvinyl alcohol hydrogel surfaces and cobalt chrome compared to the substrate materials [21,22]. The tribology of chitosan scaffolds articulating against cartilage has not been investigated.

The aim of this study was to investigate the tribological and mechanical performance of a chitosan-based OCC using a physiological porcine knee experimental simulation model [13]. The wear, damage and deformation of the cartilage surfaces were assessed following 3 h (10 800 cycles) functional simulation using geometric and scoring techniques; the stability of grafts in the recipient site was assessed; and the structure of the grafts following 3 h functional simulation was investigated using micro-computed tomography (microCT) imaging. The OCC was compared to a predicate device in current clinical use and a positive control consisting of a high modulus (stainless steel) graft implanted proud of the cartilage surface. The influence of surgical positioning of the OCC was considered by implanting the grafts either proud of the cartilage surface or inverted in addition to implanting the grafts optimally. It was hypothesized that in this experimental model, the OCC positioned optimally would function similarly to the predicate due to their comparable mechanical properties and that the high modulus, stainless steel positive control graft would lead to high wear of the opposing cartilage surfaces.

## 2. Material and methods

### 2.1. Materials

Twenty porcine knees were obtained from the right hind legs of Large White pigs aged four to six months old, within 24 h of slaughter. Throughout preparation, hydration of the cartilage surfaces was maintained using phosphate-buffered saline (PBS; MP Biomedicals LLC, UK). Once prepared, samples were stored until required at −20°C, and thawed at room temperature. Two types of synthetic osteochondral graft were investigated: (i) a novel bioresorbable chitosan/chitosan-nano-hydroxyapatite (nHA) OCC and (ii) a predicate graft in current clinical use.

The chitosan-based OCC scaffold is non-delaminating and presents a graded pore size and chemical composition with layers intended to be representative of cartilage and subchondral bone [17]. The scaffold which has a relatively slow degradation rate of 5% weight reduction in three weeks was produced as previously described and characterized by Pitrolino *et al.* [17]. The scaffolds were cut to the required diameter using a cork-borer (8.5 mm diameter) and to the appropriate length using a scalpel with 5 mm depth of the bone-like phase used throughout the study (table 1).

**Table 1.** Details of the experimental groups investigated in this study, *n* = 4 for each group.

experimental group	graft diameter (mm)	graft length (mm)
positive control (stainless steel pin 1 mm proud)	8	9
OCC flush	8.5	8 mm: cartilage-like layer 3 mm, bone-like layer 5 mm
OCC 1 mm proud	8.5	10 mm: cartilage-like layer 5 mm, bone-like layer 5 mm
OCC inverted flush	8.5	8 mm: cartilage-like layer 3 mm, bone-like layer 5 mm
predicate flush	8	graft provided as 15 mm and cut to 8 mm

The predicate was a bi-layered, resorbable scaffold with a type 1 bovine collagen cartilage layer and a β-tricalcium phosphate with polylactic acid (ratio of 80% : 20%) subchondral bone layer [7] intended for the treatment of osteochondral lesions in the knee less than 2 cm^2^ [23]. For the positive control, a stainless steel (303) cylindrical pin, 8 mm in diameter with a 100 mm radius of curvature, polished face and a radiused edge was used, a similar geometry to that of previous studies [13,14].

### 2.2. Methods

#### 2.2.1. Sample preparation and simulation model

The method for setting up the porcine tibiofemoral joint in the simulator has previously been described in detail by Liu *et al.* [12]. In brief, braces maintained the relative position of the tibia and femur in their physiological alignment during the dissection process. The femur was cemented into custom pots using a templating method to align the centre of rotation [24] of the femur with the flexion axis of the simulator. The axial force was offset medially 7% of the joint width to reflect the greater load sharing through the medial condyle during gait [25,26]. The tibia was cemented with respect to the femur using fixturing to ensure the positioning of the joint with respect to the axes of the simulator was consistent between samples. Prior to simulation, the braces used to support the joint were removed. In this model, all soft tissues and ligaments were dissected to improve access and visualization of the articulating surfaces. This simplified graft implantation and aided analysis of the cartilage surfaces; the menisci and their roots were left intact. So that the knee moved similarly to an intact knee, the function of the ligaments and soft tissue was replicated in the anterior–posterior axis simulator using springs as previously described by Liu *et al*. [12].

**Figure 1.**
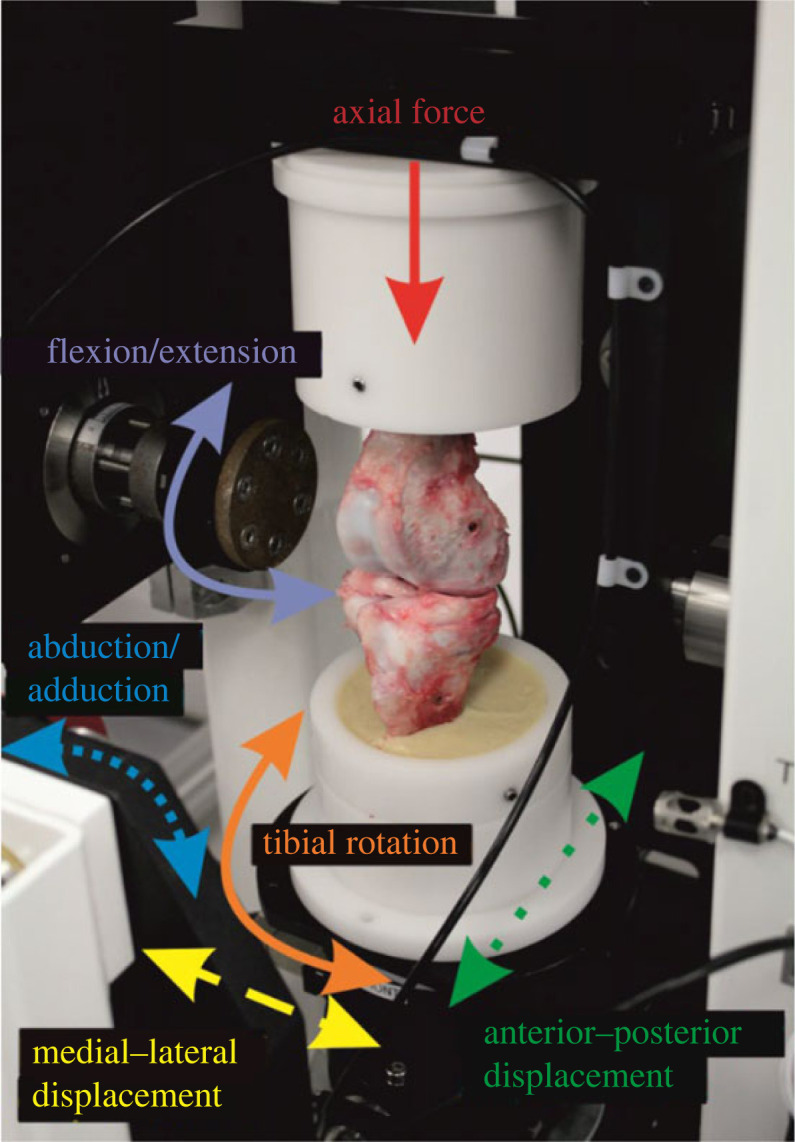
A natural porcine knee set up in a knee simulator. The gaitor (that contains the lubricant) has been removed to aid visualization of the joint. The axes of motion of the simulator are shown with solid lines representing driven axes (axial force, flexion/extension, tibial rotation), dashed lines representing either spring constrained (anterior–posterior displacement), fixed (medial–lateral displacement) or free (abduction/adduction) axes.

The study was carried out using a single station electromechanical knee simulator. The simulator (figure 1) has 6 d.f. with four controlled axes of motion, axial force, flexion/extension, tibial rotation and anterior–posterior displacement. The input profiles were consistent with Bowland *et al.* [13] but to better replicate the natural kinematics of the knee, a different spring constraint was used (20 Nm with 4 mm spring gap). The optimization of this control regime has previously been described by Liu *et al.* [27]. In brief, the input axial force and flexion/extension were based on the ISO standards for wear testing of total knee replacements but scaled for use in porcine tissue [12,25]. The input tibial rotation position was based on the natural kinematics of the knee [28] and in this study was scaled for use with porcine tissue. The anterior–posterior translation was controlled using springs, the spring rate and spring gap applied being optimized based on a previous investigation to most closely replicate the kinematics of the intact porcine knee [27].

#### 2.2.2. Study design and experimental groups

**Figure 2.**
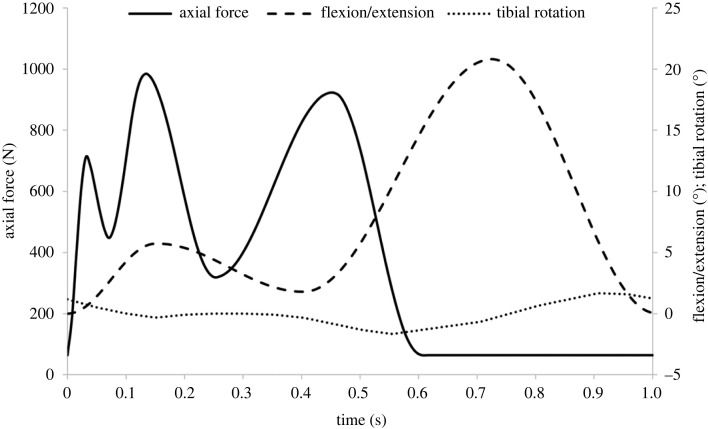
Input axial force (N), flexion/extension (°) and tibial rotation (°).

**Figure 3.**
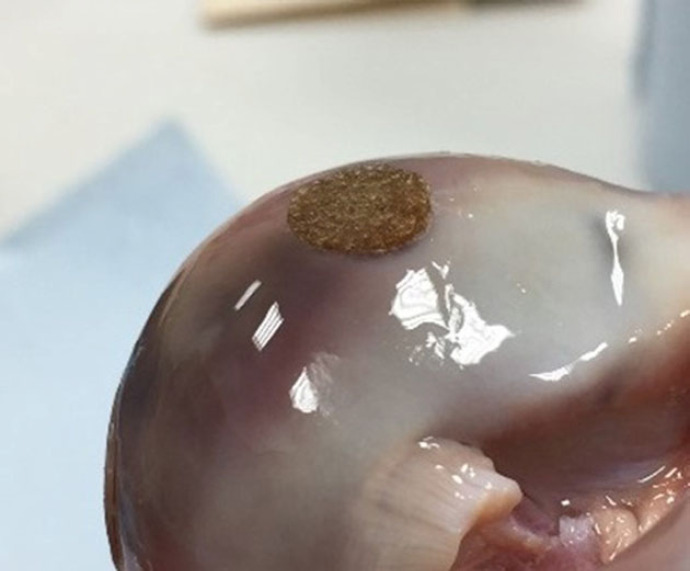
OCC implanted flush with the cartilage surface in a porcine medial condyle.

The study design was similar to previous investigations of osteochondral grafts in whole joint simulation models [13,14]. Five experimental groups, including a positive control in order to demonstrate the method was sufficiently sensitive to measure wear, were investigated as detailed in table 1, with four samples per group. Once set up, each joint was subjected to a walking gait cycle at 1 Hz (figure 2) in the simulator for 15 min (900 cycles) using 25% (v/v) bovine serum in PBS as a lubricant to determine the baseline kinematics and stability of the joint. The intervention was then introduced into the medial femoral condyle within the flexion range. The graft recipient site was created using a slot drill. All recipient sites were 8.25 mm deep and either an 8 mm diameter drill bit was used for the OCC or a 7.5 mm diameter bit for the stainless steel pins and predicate grafts to create an interference fit between the graft and recipient site. The different sizes of drill bits were used for different graft diameters (table 1). Recipient sites were drilled perpendicular to the cartilage surface and with a flat base. For the positive control group, stainless steel pins were implanted 1 mm proud of the cartilage surface. The stainless steel graft was 9 mm in length and the recipient site was drilled to a depth of 8 mm consistent with other experimental groups; this resulted in a bottomed graft with its surface proud of the surrounding cartilage (table 1). A proud stainless steel graft was used for the positive control based on previous simulation studies carried out in both the tibiofemoral and patellofemoral joints which have shown high modulus (stainless steel) grafts implanted proud of the articulating surface to result in accelerated and measurable wear, damage and deformation of the opposing cartilage surfaces [13,14]. For the OCC, prior to implantation, the graft was soaked in PBS, degassed using a syringe then implanted using a custom delivery device. The predicate graft was also pre-soaked in PBS with no active degassing step prior to implantation with the delivery device. To start to investigate the influence of surgical positioning (table 1), the OCC was either positioned optimally (OCC flush) as shown in figure 3, implanted at least 1 mm proud of the articulating surface to replicate the recipient site being under-drilled (OCC 1 mm proud) or positioned at an optimal depth but inverted to investigate whether the different properties such as the pore size, mechanics and graft composition in the cartilage-like and bone-like aspects of the graft influenced graft biomechanics and wear (OCC inverted flush). The predicate graft was implanted optimally (flush). Following graft implantation, the joint was returned to the simulator for an additional 3 h (10 800 cycles) walking gait simulation in 25% (v/v) bovine serum in PBS.

#### 2.2.3. Analysis

A visually based (by eye-examination) evaluation of the cartilage surfaces (femur, tibial plateau and superior meniscus) was performed post-test by two scorers. Macroscopic grading systems based on the International Cartilage Regeneration and Joint Preservation Society (ICRS) system [29] for scoring the femur and the tibial plateau and the Osteoarthritis Research Society International (OARSI) system for the meniscus were adapted for use in a porcine knee [30]. For the ICRS system, each femoral and tibial condyle was divided into nine regions and the most severe lesion within this region scored. Where the cartilage was normal, grade 0 was assigned; if the cartilage lesion exposed subchondral bone, a score of 4 was given. For the meniscus, a similar system was used which divided each meniscus into three zones (anterior, central and posterior) then each zone was scored. When the meniscus appeared healthy, a score of 0 was given, focal discoloration or deformation of the meniscus was assigned a score of 1 and for complete structural loss of the meniscus, a score of 4 was given. The scoring systems are summarized in table 2 and the supplementary dataset [31].

**Table 2.** Summary of the scores assigned to different damage and features on the femur, tibial plateau and meniscus.

grade	grading system for femur and tibial plateau	grading system for meniscus
0	normal: no visible damage	faint striations, no discoloration, deformation or tearing
1	nearly normal: superficial lesions including discoloration, deformation, fibrillation or abrasions	dark or thick striations, focal discoloration, presence of deformation, no or partial tearing
2	abnormal: lesions extending less than 50% cartilage depth	complete or non-complex tearing, no or low degeneration
3	severely abnormal: cartilage defects more than 50% cartilage depth	complex tearing, moderate degeneration
4	severely abnormal: deep lesions exposing subchondral bone	complete structural loss

The stability of the (positive control) graft in the femur was determined by taking replicas of the graft *in situ* using Accutrans AB silicone replicating compound (Coltene Whaledorf AG, Switzerland). Replicas were taken following implantation, then after 3 h wear simulation. The position of the (positive control) graft in the femur relative to the surrounding cartilage was then measured using an Alicona G5 optical profiler (Graz, Austria) with 5× magnification and the step height between the top of the graft and the cartilage assessed [14]. When the graft was proud, a positive step was measured and subsidence of the graft relative to the pre-test measurements led to a decrease in the measured step height. This process was carried out for the positive controls only, as when used with the open pore structure of the synthetic constructs, the silicone replicating material was seen to become embedded in the grafts. A description of the position of the grafts within the recipient site has been given since a quantitative method could not be used.

MicroCT analysis was performed to image the bone surrounding the defect and the implanted material. Scans were performed with a micro X-ray computed tomography system (Skyscan 1174, Bruker) using the following conditions: 50 kV voltage, 800 µA current, voxel resolution of 32 µm and application of a 0.50 µm aluminium filter.

Silicone replicas were also taken of the meniscus pre- and post-test and imaged using the optical profiler. The measurements were analysed to determine the change in geometry of the meniscus following simulation and the subsequent area and depth of the wear, deformation and damage scar as previously described by Bowland *et al.* [13]. Each measurement was repeated three times to check for reproducibility. The replicas were taken at the same time point following completion of the 3 h test. Further details of the wear measurement are given in the supplementary dataset [31].

The data are expressed as the mean ± s.e.m. for the cartilage grading score, the height of the (positive control) pins from the cartilage surface, the area and depth of the wear, deformation and damage. Statistical analysis was carried out in IBM SPSS Statistics for Windows, Version 26 (Armonk, NY, USA) to compare the experimental groups. Having performed a variance test, the cartilage grading score was analysed using a Kruskal–Wallis test and *post* *hoc* testing was performed using the Dunn–Bonferroni approach. Significance was taken at *p* < 0.05. The data associated with this article are openly available from the University of Leeds Data Repository [31].

## 3. Results

Each joint was initially run for 900 gait cycles to check its stability. At this time point, no wear, deformation or damage was observed on any of the articulating surfaces and all the joints were stable with no dislocations occurring.

After 3 h wear simulation with a (positive control) stainless steel pin 1 mm proud of the articulating surfaces, a region of wear, damage and deformation was evident on the menisci opposing the graft and in one sample, a small longitudinal meniscal tear was visible in zone 3 [32] of the meniscus. Chondral lesions (ICRS grade 1–2) were evident on the tibial plateau of two samples. There was no evidence of subsidence of the stainless steel pin in the femur. Analysis of the position of the pin showed a mean protrusion of 1.24 ± 0.06 mm and 1.19 ± 0.05 mm for the pre- and post-test measurements, respectively.

After 3 h simulation for the experimental group with OCCs implanted flush with the femur, there was no visible wear, damage or deformation on the meniscus of three samples; however, one meniscus exhibited a region of circular deformation opposing the graft site. Isolated scratches were visible on the tibial plateau (ICRS grade 1) and subsidence/compression of the graft was evident resulting in the surface of the graft sitting approximately 0.5 mm below the cartilage surface. Implanting the OCC 1 mm proud of the surface resulted in visible discoloration of the meniscus in three of the four samples and isolated (ICRS grade 1) scratches on the tibial plateau. The grafts, which were initially positioned 1 mm proud of the cartilage surface, were, at 3 h, flush with the cartilage. Implanting the grafts flush with the cartilage surface but inverted resulted in regions of discoloration on the meniscus in three of the four samples and isolated scratches (ICRS grade 1) on the tibial plateau. After 3 h simulation, the graft was below the level of the surrounding cartilage surface. Subsidence of the inverted graft appeared to be greater than that of the graft in the correct orientation.

Predicate samples were implanted optimally with the surface of the graft flush with the surrounding cartilage. Following 3 h simulation, isolated scratches were visible on the menisci articulating against the graft in all samples, and a high density of fine (ICRS grade 1) scratches were visible on the tibial plateau. On the femur, scratching/scuffing was visible on the cartilage surrounding the graft recipient site (ICRS grade 1). Loss of structural integrity and change in the surface topography of the graft occurred in two of the four samples with a section of the graft becoming detached from one sample.

**Figure 4.**
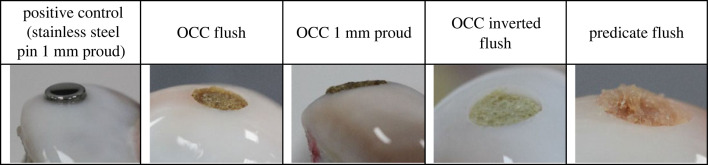
Representative images of the grafts implanted in porcine medial femoral condyles following 3 h wear simulation.

**Figure 5.**
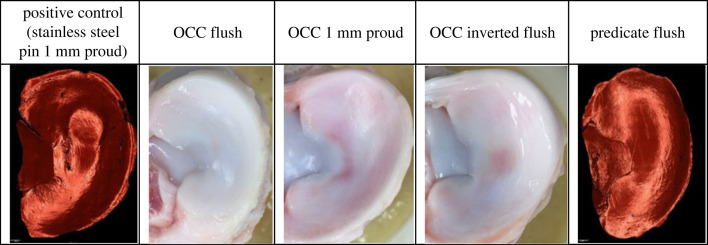
Representative images of the meniscus opposing the graft following 3 h wear simulation. When discoloration of the meniscus was visible, optical photographs have been used; for changes in surface topography, measurements of the meniscus taken from replicas of the cartilage surfaces have been provided.

Representative images of the grafts and menisci at the conclusion of the study are shown for each experimental group in figures 4 and 5.

**Figure 6.**
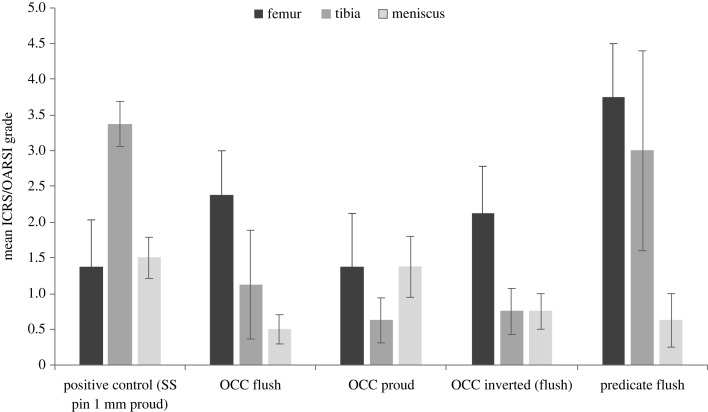
Mean (± s.e.m.) cartilage grade on the femoral condyles and tibial plateau measured on the ICRS scale, and the menisci measured on the OARSI scale, *n* = 4.

Mean cartilage damage scores on the femur, meniscus and tibial plateau are shown in figure 6. There was no significant difference in mean cartilage score between the experimental groups on the femur (*p* = 0.23), although for the knees with a predicate graft, the mean score was almost twice that of the knees with the OCC. The mean cartilage score on the femur for the OCC was similar irrespective of the surgical positioning of the graft. On the meniscus, the region of deformation on the menisci articulating against stainless steel pins 1 mm proud (positive controls) and the discoloration of the meniscus articulating against the OCC proud grafts resulted in higher scores than for the other OCC groups and predicate grafts, although there was no significant difference between the experimental groups (*p* = 0.06). For the tibia, the grading scores were highest in the positive control group and the predicate group; however, there was no significant difference between the experimental groups (*p* = 0.17).

Analysis of the area and depth of the wear, deformation and damage scar was carried out from Accutrans replicas of the menisci using the Alicona G5 optical profiler. For the OCC, OCC proud, OCC inverted and predicate groups, the wear, deformation and damage were below a measurable resolution for the technique used. For the positive controls, there was a clearly defined circular region of deformation on the anterior aspect of the meniscus (figure 5); the mean depth of the wear, damage and deformation scar was 451.9 ± 125.1 µm and the wear area was 60.9 ± 11.2 mm^2^. This was the only experimental group in which a measurable wear, damage and deformation scar could be measured.

**Figure 7.**
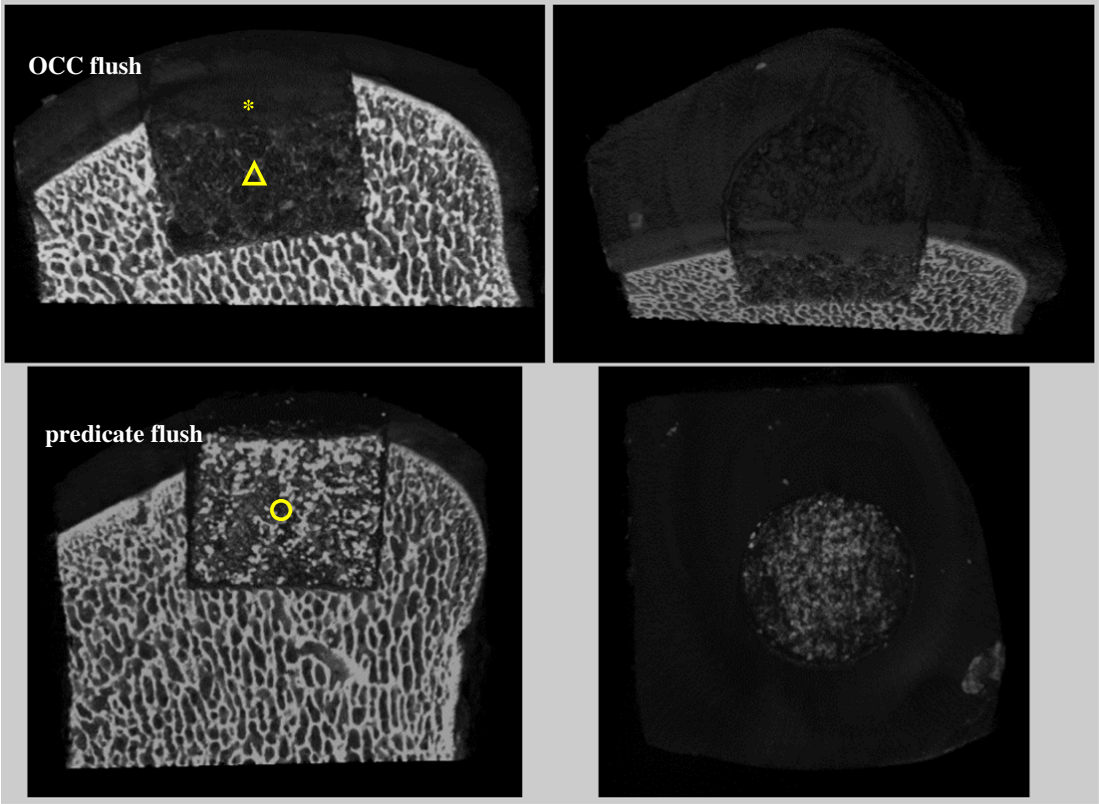
Representative microCT images of grafts following 3 h simulation. Top: OCC flush (with cartilage-like (*) and bone-like (Δ) regions highlighted). Bottom: predicate (indicated by ◯). The images on the left and right show the lateral and top views from the same samples, respectively. OCC recipient site 8 mm in diameter; predicate recipient site 7.5 mm in diameter.

Representative images of the OCC flush and predicate grafts taken using microCT are shown in figure 7. The OCC graft expanded to fit within the recipient site with minimal void around the graft; a radiolucent line was visible around the predicate graft (circle). The porous structure was visible in the predicate graft, whereas the OCC had a more trabecular bone-like structure with the ‘bone' (triangle) and ‘cartilage' (asterisk) layers being clearly defined as shown in figure 7.

## 4. Discussion

This is the first study to investigate the functional performance of a chitosan OCC implanted in a porcine knee model applying physiological loading and motions. The wear, damage and deformation of the opposing cartilage surface were assessed, along with the stability of the graft (subsidence) in the recipient site. The chitosan OCC was compared to a predicate graft in current clinical use and the influence of surgical positioning of the OCC was investigated by implanting the graft either optimally, inverted or proud of the cartilage surface. This is the first time that synthetic osteochondral grafts have been studied in this type of *in vitro* pre-clinical investigation. This simulation model can be carefully controlled and can provide insight into the tribological and biomechanical performance of osteochondral grafts over extended gait cycles, which may not be obtained through *in vivo* studies, and can be used to reduce and support animal studies.

### 4.1. Wear, damage and deformation following functional simulation

After 3 h wear simulation, the positive control samples showed a clear region of wear, damage and deformation on the meniscus, the depth of the wear scar (0.45 ± 0.40 mm) being similar to that previously measured by Bowland *et al.* [13] (0.58 mm). Owing to the different diameter grafts and variations in the simulation systems used in the two investigations, the depth of the wear scar rather than its volume was deemed to be the most appropriate comparator. The damage seen on the menisci was likely caused by a combination of factors including the elevated contact pressure due to positioning the graft proud of the cartilage surface, the high modulus of the pin and its geometry [33]. For all the synthetic grafts investigated, the wear, damage and deformation were low, below a threshold measurable using optical profilometry, and the position (flush versus proud) of the OCC graft did not appear to influence the wear. The lower modulus of the synthetic grafts compared to the stainless steel pin used in the positive controls or an allograft [13,14] likely contributed to the low wear. The modulus of cancellous bone has been measured ranging from 0.1 to 0.5 GPa [34,35] and stainless steel in excess of 150 GPa. Compressive testing of wet chitosan composite scaffolds has shown a modulus ranging from approximately 0.01 to 5 MPa depending on the graft composition and proportion of chitosan in the scaffold [16,17]. Previous mechanical testing of the bi-layered chitosan scaffold used in this study showed a compressive modulus of 1.6 MPa when tested dry and a significantly lower modulus when tested following 60 min immersion in distilled water (0.013 MPa) [17].

Although wear was not measurable, visual examination of the articulating surfaces and scoring of the damage highlighted differences between the experimental groups. Implantation of the predicate grafts resulted in visible scratching on the meniscus; whereas for the OCC implanted either proud or inverted, there was discoloration of the meniscus where the graft articulated against. This resulted in similar grading scores of the menisci post-test but the mechanism for the damage differed depending on the grafts. Discoloration of the meniscus was not apparent in the positive control samples, OCC flush or predicate experimental groups. It is unclear whether discoloration of the meniscus indicated any loss of structural integrity in this experimental model; however, *in vivo*, tissue discoloration and deformation can be an early sign of osteoarthritis and may present as a precursor to tearing [36]. In the positive control samples, cartilage lesions were also visible on the tibial plateau, this mode of damage having not been previously reported in similar experimental studies [13]. Similar ‘kissing lesions' on the tibial plateau have been observed in the clinic when osteochondral grafts have been implanted proud of the cartilage surface and may result in chondral defects in the tibial plateau, with pain and changes in biomechanics due to the graft catching as the joint articulates [37]. It is thought that the larger diameter graft used in this study (8 mm in diameter compared to 6.5 mm in diameter used by Bowland *et al*. [13]) may have been a contributing factor in the formation of these lesions. Consistent damage to the tibial plateau was not seen in any of the OCC groups; however, all samples with a predicate graft showed isolated scratches on the tibial plateau. The cause of these scratches is unknown; however, it is hypothesized that material released from the graft may have caused the scratching. For scratching to occur on the cartilage, the hardness of the released particles must be greater than that of the cartilage [38]; the hardness of particles potentially released from the predicate graft is unknown. However, due to the high volume of lubricant used (approx. 1 l) and the potentially small volumes of particulate debris, it was not possible to analyse the particles in the lubricant. On the femur, the most consistent damage occurred in the predicate group where scratching was evident around the graft site; the mode of damage was thought to be similar to that for the tibial plateau and related to potential release of particulate material from the grafts.

### 4.2. Graft stability following functional simulation

There are a number of factors which may influence the primary stability of grafts in the recipient site including the preparation of the recipient site and the relative modulus of graft and host tissue [39,40]. The protrusion of the positive control graft was assessed pre- and post-test and remained similar over the duration of the study. In previous investigations of osteochondral allografts and stainless steel pins implanted in the tibiofemoral joint, similar results have been reported [13]. Irrespective of whether implanted flush, proud or inverted, all the OCCs were seen to subside/compress *in situ* with the inverted grafts appearing to subside more than those implanted in the correct orientation. MicroCT imaging of the optimally positioned graft showed the OCC to sit within the recipient site without visible radiolucent lines due to oversizing of the graft compared to the recipient site. From the images, it seems unlikely that the graft subsidence was caused by a change in the recipient site the base of which remained flat bottomed and more probable that compression of the graft occurred. It was not possible to determine from the imaging whether compression occurred equally along the length of the graft or was more pronounced in either the bone-like or cartilage-like region. Previous mechanical testing of the graft in its hydrated state has demonstrated its ability to recover its shape when compressed longitudinally up to 70% compressive strain and when radially compressed by approximately 30% for delivery through a cannula [17]. There are a number of possibilities for why when implanted in the simulation model the graft appeared not to fully recover its geometry. These include the high (10 800) number of repetitive cycles, the complex loading and motion of the knee joint and the unknown interactions between the inside walls of the recipient site. No delamination of the OCC graft was observed, consistent with previous tensile mechanical tests [17]. It is not known whether the grafts were compressed or whether any material may have been lost from the graft surface. Subsidence of osteochondral grafts *in vivo* is not desirable and may lead to the formation of fibrocartilage with inferior biomechanics to native cartilage [41–43]. Subsidence of the predicate grafts was not apparent despite radiolucent lines being visible around the graft, but after 3 h simulation there was a visible change in two grafts which appeared to suffer from a loss of structural integrity with failure or delamination occurring in one graft. This change in the graft surface made determining potential graft subsidence difficult. The preparation technique used here for all grafts involved soaking in PBS prior to implantation; however, the predicate surgical preparation includes soaking the graft in blood prior to implantation. It is not known whether the preparation technique may have influenced the fragmentation of the graft. The primary stability of the graft is important as it may influence long-term bone integration [39,40], a parameter that will require complementary animal studies to understand how the graft integrates with host tissue.

### 4.3. Limitations

There were several limitations associated with this study which may influence the interpretation of these findings. Firstly, the same implantation technique of the synthetic grafts was used for the OCC and for the predicate grafts, although the instructions for the implantation of the predicate grafts did not specify the condition of the base of the recipient site or its diameter. There were also limitations as to assessment of the graft following wear simulation as a qualitative assessment was carried out; this means that it is not known whether the compression/subsidence of the grafts during simulation might have resulted in loss of material. If particulate material was to enter the joint space, there may be acceleration of cartilage wear and damage through a third body wear mechanism, and the scratches which were visible particularly for the predicate group suggest this wear mode may have occurred in this preliminary study. It was not possible to assess the pre- and post-test position of the grafts in the recipient site using the replication material because preliminary trials showed the replication material to enter the open porous structure of the scaffolds. This could have influenced graft performance if used, and there was also potential for the replication material to unseat the graft from the recipient site, and therefore the procedure was not performed. The CT scans showed the position of the grafts within the recipient site; however, these scans were taken a significant time after the study and following freezing and defrosting of the samples. Previous investigations of the OCC have demonstrated its ability to restore its geometry after compression [16,17] so the position of the graft when scanned will likely be different from its position following the simulation. Osteochondral grafts aim to restore the biomechanics and tribology of the joint, but it is not known whether these grafts were able to restore the native contact pressure and contact area within the joint. Future studies could further investigate contact pressure with the graft *in situ*.

There were also limitations with the assessment of wear on the meniscus. The use of an optical profilometry geometric technique to assess the change in geometry of the meniscus after 3 h simulation measured a combination of wear (loss of material), damage and permanent deformation. It was not possible to decouple wear, damage and deformation so it was important that the timepoint at which the replicas were taken following simulation was the same for all samples. In most samples, the wear on the meniscus was below a threshold which could be measured volumetrically and the technique used could not differentiate between grafts or their position. Future studies may consider microscopic rather than macroscopic damage perhaps considering techniques such as histology. There were limitations with the simulation system including only investigating the medial condyle consistent with Bowland *et al.* [13]. Although the force passing through the medial condyle is greater than the lateral, it is not known whether introducing the intervention into the lateral condyle, which may have an increased anterior–posterior translation, may generate more damage to either the graft or the meniscus. The simulation was also limited in that only a single condition was investigated which aimed to replicate a walking gait and the use of porcine tissue meant that the input forces and motions were likely lower than those which would be applied in the human knee. In addition, this was a short-term study investigating the functional performance of one composition of chitosan osteochondral graft prior to tissue integration and remodelling. Previous studies have shown poor tissue integration of one formulation of a chitosan-based scaffold when used as an osteochondral graft, in both rabbit and sheep models [44], which highlights the need to fully characterize the grafts prior to clinical adoption.

Finally, the sample size investigated for this study was limited to four samples per experimental group. A sample number consistent with previous investigations [13] was chosen based on the practicalities of the time involved in setting up and running the simulation studies, and the need to investigate five different experimental groups. The sample size was sufficiently large to demonstrate the wear, deformation and damage to the meniscus in the positive controls, the subsidence of the OCC and the deterioration of the predicate graft surface across the samples studied. This shows that despite the relatively small sample size, the method was able to discriminate between interventions.

## 5. Conclusion

Implanting a new chitosan-based OCC either optimally (flush), inverted or proud of the cartilage surface resulted in minimal wear, damage and deformation of the meniscus and positioning had no influence on cartilage wear; however, all grafts were seen to subside below the cartilage surface after 3 h simulation. There were no obvious signs of OCC material failure. This functional performance assessment for grafts prior to tissue integration and remodelling can provide key information on implant materials ahead of pre-clinical investigations in animal models.

## Data Availability

The data associated with this paper are openly available through the University of Leeds data repository: https://doi.org/10.5518/1406 [31].
